# Paradoxical Acute Hypoxemic Deterioration During Treatment of Bilateral Bronchopneumonia: A Case Report

**DOI:** 10.7759/cureus.115567

**Published:** 2026-08-31

**Authors:** Cansu Ak

**Affiliations:** 1 Department of Pulmonology, MBAL Dr. Nikola Vasiliev, Kyustendil, BGR

**Keywords:** bronchopneumonia, community-acquired pneumonia, drug-related reaction, hypoxemic respiratory failure, paradoxical deterioration

## Abstract

Community-acquired pneumonia may occasionally be associated with unexpected respiratory deterioration despite apparent clinical, laboratory, and radiological improvement. We report a case of a previously healthy adult male who presented with progressive dyspnea, persistent cough, generalized weakness, malaise, and low-grade fever. Initial chest radiography showed no significant abnormalities despite marked respiratory symptoms, whereas chest computed tomography revealed bilateral bronchopneumonia with multiple centrilobular nodules and patchy ground-glass opacities. The patient was treated with intravenous meropenem, systemic corticosteroids, inhaled therapy, supplemental oxygen, and supportive care, with gradual improvement in inflammatory markers. On hospital day three, however, he developed sudden severe hypoxemic respiratory failure shortly after antimicrobial administration, requiring intensive care unit admission and high-flow nasal cannula oxygen therapy. Urgent CT pulmonary angiography excluded pulmonary embolism, follow-up imaging demonstrated partial regression of the pulmonary infiltrates, and cardiological evaluation showed no evidence of acute cardiac pathology. Meropenem was replaced with levofloxacin because a drug-related reaction could not be completely excluded. The patient’s respiratory status subsequently improved rapidly with supportive treatment, and he was discharged without residual respiratory insufficiency. This case demonstrates that severe transient hypoxemic deterioration may occur during apparently successful treatment of bilateral bronchopneumonia and emphasizes the importance of promptly excluding pulmonary embolism, acute cardiac disease, drug-related reactions, and other potentially life-threatening causes of respiratory worsening.

## Introduction

Community-acquired pneumonia (CAP) remains a leading cause of hospitalization and infectious disease-related morbidity and mortality worldwide [1-3]. Although chest radiography is generally the first-line imaging examination, its sensitivity for detecting community-acquired pneumonia is limited compared with CT, particularly early in the disease course or when pulmonary abnormalities are subtle. Chest computed tomography can identify infiltrates that are not visible on chest radiography, particularly ground-glass opacities and centrilobular nodules [4-6].

Although most patients show gradual clinical improvement after appropriate antimicrobial therapy, unexpected respiratory deterioration may occur despite favorable clinical, laboratory, and radiological findings. Patients who fail to respond as expected or who deteriorate during treatment require prompt and systematic reassessment to identify potentially reversible causes of treatment failure or alternative diagnoses [7]. Such deterioration requires urgent reassessment to distinguish progression of infection from other potentially serious conditions. Pulmonary embolism, acute cardiac disease, acute respiratory distress syndrome, organizing pneumonia, and drug-induced pulmonary injury should be considered in the differential diagnosis [8-14]. Such transient hypoxemic deterioration despite apparent clinical and radiological improvement may be clinically important because it can prompt urgent reassessment for alternative, potentially life-threatening diagnoses.

In this report, we describe a patient with CT findings consistent with bilateral bronchopneumonia who developed abrupt severe hypoxemia despite improving inflammatory markers and radiological regression. This case highlights the diagnostic challenge posed by acute respiratory deterioration occurring during an apparently favorable therapeutic response.

## Case presentation

A previously healthy adult male presented to the emergency department with a several-day history of progressively worsening dyspnea, persistent non-productive cough, generalized weakness, malaise, and low-grade fever, with a maximum recorded temperature of 37.5°C. He denied recent travel, known exposure to tuberculosis, or contact with individuals with respiratory infections. His medical history was unremarkable, with no history of chronic respiratory disease, cardiovascular disease, diabetes mellitus, chronic kidney disease, malignancy, or immunosuppressive disorders. He was not receiving any regular medication and had no known drug allergies.

On admission, the patient appeared mildly distressed because of dyspnea but remained alert and hemodynamically stable. Pulmonary auscultation revealed bilateral fine inspiratory crackles, whereas cardiovascular examination was unremarkable. Initial laboratory investigations demonstrated elevated inflammatory markers consistent with an acute infectious process. Cardiac biomarkers were within the normal reference range, with no laboratory evidence suggestive of acute myocardial injury. Before the initiation of antibiotic therapy, blood cultures, sputum culture, respiratory viral PCR testing, and influenza/COVID-19 testing were performed, and all results were negative. No causative pathogen was identified despite this microbiological evaluation.

A posteroanterior chest radiograph performed on admission showed no focal consolidation or other radiographic abnormalities sufficient to explain the severity of the patient’s respiratory symptoms. Because of the discrepancy between the clinical findings and the nearly normal chest radiograph, a non-contrast chest computed tomography (CT) scan was obtained for further evaluation (Figure 1, panel A). Chest CT demonstrated multiple bilateral centrilobular nodules accompanied by patchy ground-glass opacities involving both lungs, predominantly affecting the left upper lobe and the right middle lobe. These findings were consistent with bilateral bronchopneumonia. No pleural effusion, cavitation, or significant mediastinal lymphadenopathy was identified (Figure 1, panels B and C).

**Figure 1:**
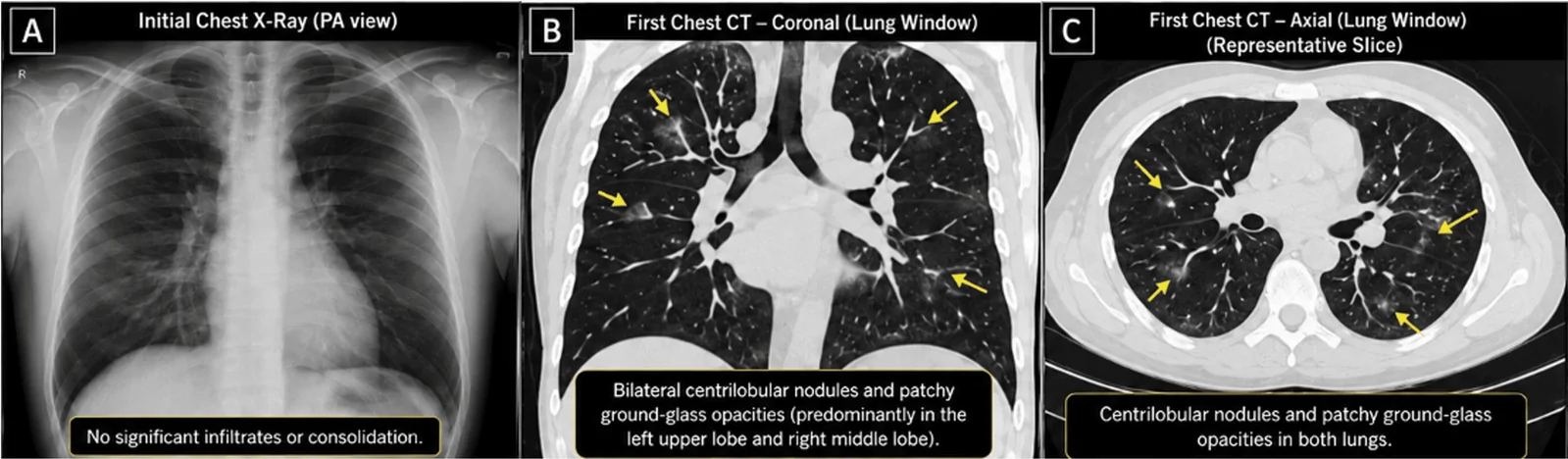
Initial chest imaging findings. (A) Initial posteroanterior chest radiograph demonstrating no definite radiographic evidence of pneumonia. (B, C) Coronal and axial chest CT images (lung window) demonstrating bilateral centrilobular nodules and patchy ground-glass opacities (arrows), consistent with bilateral bronchopneumonia.

The patient was admitted to the department of pulmonology and was started on intravenous meropenem (1 g every 8 h), systemic methylprednisolone, inhaled bronchodilator therapy, supplemental oxygen, and supportive treatment. During the first two days of hospitalization, his respiratory symptoms remained clinically stable. Serial inflammatory markers did not show a uniform decline; CRP initially increased from 10.43 to 17.55 mg/L before subsequently declining during the later hospital course. The timeline of the clinical events is shown in Figure 2, panel A.

**Figure 2:**
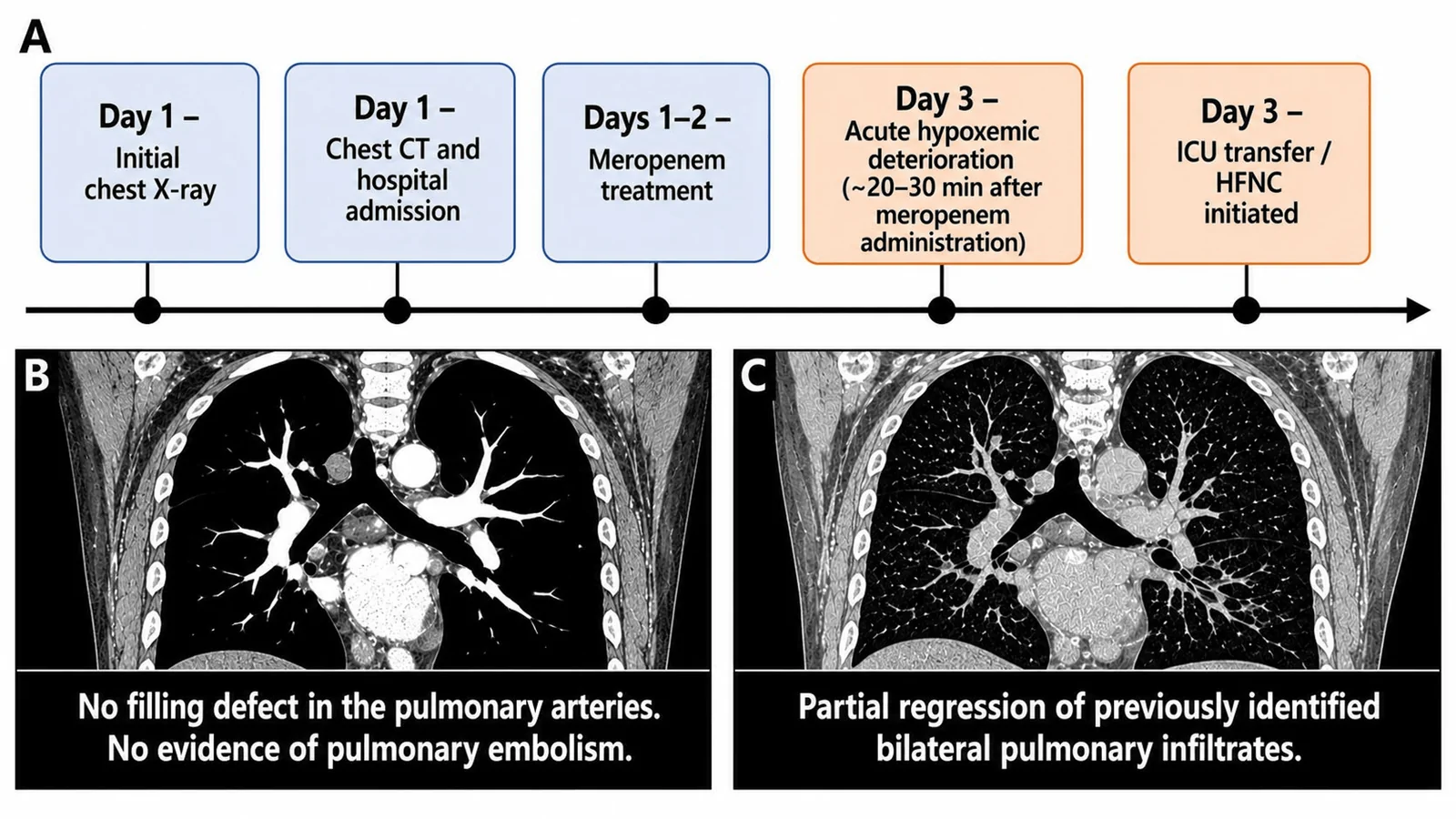
Clinical course and follow-up imaging. (A) Timeline of the patient’s clinical course. (B) CT pulmonary angiography demonstrating no evidence of pulmonary embolism. (C) Follow-up chest CT demonstrating partial regression of the previously identified bilateral pulmonary infiltrates. HFNC: high-flow nasal cannula

Unexpectedly, on the third hospital day, approximately 20-30 min after the scheduled administration of intravenous meropenem, the patient developed sudden chest tightness followed by rapidly progressive dyspnea and severe hypoxemia, with peripheral oxygen saturation (SpO₂) falling to 83% on room air. His respiratory status deteriorated rapidly, necessitating immediate assessment by the resuscitation team and urgent transfer to the intensive care unit.

High-flow nasal cannula (HFNC) oxygen therapy was initiated for acute hypoxemic respiratory failure, resulting in gradual improvement in oxygenation. Given the abrupt onset of respiratory deterioration, acute pulmonary embolism was considered the primary differential diagnosis. Urgent CT pulmonary angiography demonstrated no evidence of pulmonary embolism or major vascular obstruction (Figure 2, panel B). Furthermore, follow-up CT imaging revealed partial regression of the previously identified pulmonary infiltrates compared with the initial examination (Figure 2, panel C).

A comprehensive cardiological evaluation, including transthoracic echocardiography, demonstrated preserved biventricular systolic function without evidence of acute heart failure, significant valvular disease, or other structural cardiac abnormalities that could explain the patient’s sudden clinical deterioration. Serial laboratory investigations likewise failed to identify an alternative cause for the hypoxemic episode, while inflammatory markers subsequently declined during the remainder of the hospitalization.

Although there was no definitive clinical evidence of an immediate hypersensitivity reaction, meropenem was discontinued because a drug-related pulmonary reaction could not be completely excluded. Antimicrobial therapy was subsequently changed to intravenous levofloxacin, while supportive respiratory care was continued.

The patient’s clinical condition improved progressively over the following days. HFNC oxygen therapy was successfully discontinued, and he was transferred back to the pulmonology ward after stabilization. Continued clinical improvement was accompanied by normalization of inflammatory markers and partial radiological regression of the pulmonary infiltrates.

The patient was discharged home in good general condition with normal oxygen saturation while breathing room air and without residual respiratory insufficiency. At outpatient pulmonology follow-up, he remained clinically well, and no recurrent episodes of respiratory deterioration were reported. Serial laboratory findings, arterial blood gas analyses, imaging findings, treatment interventions, differential diagnoses, and the chronological clinical course are summarized in Tables 1-7.

**Table 1:** Serial laboratory findings during hospitalization.

Parameters	Reference range	Day 1	Day 3	Day 5 (ward)	Day 5 (ICU)	Day 8	Day 11
WBC (×10⁹/L)	4-10	15.93	12.70	9.76	9.97	11.71	9.82
Neutrophils (%)	40-75	78.2	76.6	47.9	71.5	48.5	80.7
Lymphocytes (%)	20-45	17.2	18.2	44.7	23.6	45	14.9
Hemoglobin (g/L)	135-175	153	148	133	148	152	154
Platelets (×10⁹/L)	150-400	328	313	337	350	312	377
CRP (mg/L)	<5	10.43	17.55	7.55	10	-	0.36

**Table 2:** Arterial blood gas analysis. HFNC: high-flow nasal cannula

Parameters	Day 3	Day 3 (hypoxemia)	Day 3 (HFNC)
pH	7.42	7.45	7.45
PaO₂	8.44	6.86	7.16
Oxygen saturation (%)	92.7	88.7	89.1
Standard HCO₃	21.3	20.7	20.8
Base excess	-5.8	-6.8	-6.9

**Table 3:** Summary of serial serum biochemical parameters measured during hospitalization. AST: aspartate aminotransferase; ALT: alanine aminotransferase

Parameters	Day 3	Day 5	Day 11
Glucose	5.6	4.0	-
Creatinine (µmol/L)	66.4	64.7	64.9
AST (U/L)	41	53	-
ALT (U/L)	83	84	-
Sodium (mmol/L)	141.1	144	-
Potassium (mmol/L)	4.64	4.38	-
Troponin	Negative	Negative	-
D-dimer	-	130.29	64.74

**Table 4:** Summary of chest imaging findings obtained during the initial assessment and subsequent clinical deterioration.

Day	Imaging	Findings
Day 1	Chest radiograph	No definite radiographic evidence of pneumonia despite respiratory symptoms
Day 1	Chest CT	Bilateral centrilobular nodules and multifocal patchy ground-glass opacities consistent with bilateral bronchopneumonia
Day 3	CT pulmonary angiography	No evidence of pulmonary embolism
Follow-up	Chest CT	Partial regression of bilateral pulmonary infiltrates

**Table 5:** Summary of the major treatment interventions and clinical management during hospitalization. HFNC: high-flow nasal cannula; CTPA: computed tomography pulmonary angiography

Hospital day	Treatment/intervention
Day 1	Admission; IV meropenem (1 g q8h); IV methylprednisolone; inhaled bronchodilator; supplemental oxygen
Day 3	Acute hypoxemia (SpO₂ ≈83%); ICU admission; HFNC initiated; CTPA performed
Day 3	Meropenem discontinued; IV levofloxacin initiated
Following days	Progressive clinical improvement
Discharge	Complete clinical recovery

**Table 6:** Differential diagnosis of acute respiratory deterioration.

Differential diagnosis	Diagnostic evaluation	Final assessment
Progression of pneumonia	Serial CT imaging	Unlikely
Pulmonary embolism	CT pulmonary angiography	Excluded
Acute coronary syndrome	Troponin and ECG	Excluded
Acute heart failure	Echocardiography	Excluded
Drug-induced pulmonary reaction	Clinical assessment	Not confirmed
Secondary organizing pneumonia	Radiological follow-up	Considered but unlikely

**Table 7:** Chronological clinical course. HFNC: high-flow nasal cannula; CTPA: computed tomography pulmonary angiography

Timeline	Clinical event	Key findings/management
Before admission	Symptom onset	Progressive dyspnea, dry cough, malaise, weakness, low-grade fever
Emergency department	Initial assessment	Stable, inspiratory crackles, elevated inflammatory markers
Admission	Chest CT	CT findings consistent with bilateral bronchopneumonia
Hospital day 1	Initial treatment	Meropenem, methylprednisolone, bronchodilator, oxygen therapy
Hospital day 3	Acute deterioration	Chest tightness; SpO₂ ≈83%
Hospital day 3	ICU management	HFNC initiated; CTPA negative for PE
Hospital day 3	Treatment modification	Meropenem discontinued; IV levofloxacin initiated
Following days	Recovery	Clinical and radiological improvement
Discharge	Outcome	Normal oxygen saturation on room air
Follow-up	Final outcome	Complete recovery without recurrence

## Discussion

Community-acquired pneumonia (CAP) remains one of the leading causes of hospitalization and infectious disease-related mortality worldwide despite substantial advances in antimicrobial therapy and supportive care. Although most patients experience gradual clinical improvement following appropriate treatment, unexpected respiratory deterioration during hospitalization represents an important diagnostic and therapeutic challenge. In such situations, clinicians must rapidly distinguish progression of infection from other potentially life-threatening conditions, including pulmonary embolism, acute respiratory distress syndrome (ARDS), cardiogenic pulmonary edema, aspiration, and drug-induced pulmonary toxicity.

The present case illustrates an unusual clinical course characterized by abrupt hypoxemic respiratory failure despite otherwise favorable clinical and radiological findings. Inflammatory biomarkers initially increased and subsequently declined, while follow-up CT imaging demonstrated partial regression of the pulmonary infiltrates. Taken together, these findings made progressive uncontrolled pulmonary infection less likely as the sole explanation for the patient’s sudden deterioration, although infection could not be completely excluded. This discrepancy between the improving clinical and radiological course and the acute respiratory decompensation emphasizes the importance of considering alternative diagnoses and mechanisms when patients unexpectedly deteriorate during antimicrobial therapy.

An important feature of this case was the marked discrepancy between the initial chest radiograph and chest CT findings. Although chest radiography remains the first-line imaging modality for patients with suspected pneumonia, its sensitivity is limited, particularly during the early stages of disease or in cases with subtle parenchymal abnormalities. Chest CT has been shown to detect pulmonary infiltrates with considerably greater sensitivity, especially in patients presenting with ground-glass opacities or centrilobular nodules. Our patient’s initially unremarkable chest radiograph, despite bilateral pulmonary abnormalities on CT, reinforces the importance of further imaging when clinical suspicion remains high despite inconclusive radiographic findings [1-4].

Several mechanisms may be considered in relation to the transient deterioration observed in this patient; however, none could be definitively established. One possible, although unproven, explanation is a transient inflammatory response occurring despite apparent clinical and radiological improvement. Paradoxical inflammatory reactions have been described in a variety of infectious diseases and have been associated with dysregulated immune activation during pathogen clearance. However, this phenomenon has not been extensively investigated in bacterial bronchopneumonia, and its relevance to the present case remains speculative. It therefore represents only one possible biological explanation for transient respiratory deterioration during an otherwise improving clinical course [8,15-19].

Secondary organizing pneumonia should also be considered in the differential diagnosis. Organizing pneumonia may develop after bacterial or viral respiratory infections and can present with worsening respiratory symptoms despite appropriate antimicrobial therapy. However, the patient’s rapid clinical improvement and subsequent radiological regression, without a prolonged clinical course, made organizing pneumonia less likely, although a transient inflammatory process cannot be entirely excluded [9-11].

Drug-induced pulmonary injury represented another important diagnostic consideration. Because respiratory deterioration occurred shortly after intravenous meropenem administration, an immediate drug-related reaction was considered. Nevertheless, the absence of skin manifestations, bronchospasm, circulatory instability, eosinophilia, or persistent respiratory impairment did not support classical anaphylaxis or provide definitive evidence of drug-induced lung disease. Consequently, although meropenem was discontinued and antimicrobial therapy was changed as a precautionary measure, a direct causal relationship between meropenem administration and the observed clinical deterioration could not be established [10-12].

Pulmonary embolism was the most critical diagnosis requiring urgent exclusion because of the patient’s abrupt onset of hypoxemia and chest discomfort. CT pulmonary angiography demonstrated no evidence of pulmonary embolism, while transthoracic echocardiography showed no evidence of acute cardiac dysfunction. These findings substantially narrowed the differential diagnosis and supported continued supportive management while other potential causes of the transient hypoxemic episode were considered [5,6,12-14].

An additional important feature of this case was the favorable response to high-flow nasal cannula (HFNC) oxygen therapy. HFNC has become an established component of supportive management for acute hypoxemic respiratory failure by improving oxygenation, decreasing anatomical dead space, reducing the work of breathing, and potentially avoiding invasive mechanical ventilation. The patient’s rapid stabilization following HFNC demonstrates the potential value of early non-invasive respiratory support in carefully selected patients experiencing sudden deterioration during treatment of community-acquired pneumonia [20].

This report emphasizes several important clinical lessons. First, a normal or minimally abnormal chest radiograph does not exclude clinically significant pneumonia when clinical suspicion remains high. Second, sudden respiratory deterioration during apparently appropriate antimicrobial therapy should prompt immediate reassessment for pulmonary embolism, acute cardiac disease, drug-related adverse reactions, progression of infection, and other alternative diagnoses rather than being attributed automatically to a single mechanism. Finally, early recognition, prompt diagnostic reassessment, and timely supportive respiratory management may facilitate complete recovery even after severe transient hypoxemic respiratory failure.

The individual components of this presentation, including pneumonia detectable on CT despite an initially negative or minimally abnormal chest radiograph and transient inflammatory deterioration during treatment, have been described in the literature [1-4,8,15-19]. However, the concurrence of these findings with sudden severe hypoxemia, exclusion of pulmonary embolism by CT pulmonary angiography, subsequent radiological regression, and complete clinical recovery is less well characterized. Additional well-documented cases may help improve understanding of the mechanisms underlying unexpected respiratory deterioration during treatment of bacterial pneumonia and may further inform future diagnostic and therapeutic strategies.

As a single-patient case report, this study has inherent limitations. Microbiological confirmation of the causative pathogen was unavailable, and the precise mechanism underlying the transient hypoxemic deterioration could not be definitively established. Nevertheless, the detailed clinical, laboratory, imaging, and follow-up findings provide insight into the diagnostic challenges and management considerations associated with unexpected respiratory deterioration during treatment of bilateral bronchopneumonia.

## Conclusions

This case demonstrates that severe transient hypoxemic respiratory deterioration may occur during apparently appropriate treatment in a patient with CT findings consistent with bilateral bronchopneumonia, even as inflammatory markers subsequently decline and follow-up imaging demonstrates partial regression of pulmonary infiltrates. Unexpected clinical worsening should prompt immediate reassessment for pulmonary embolism, acute cardiac disease, drug-related pulmonary reactions, progression of infection, and other potentially life-threatening conditions. The precise mechanism of the acute hypoxemic deterioration in this case could not be definitively established.

Early recognition, comprehensive diagnostic reassessment, and timely supportive respiratory management, including high-flow nasal cannula oxygen therapy when indicated, are important in the management of acute hypoxemic deterioration. This case also emphasizes the value of chest computed tomography in patients with high clinical suspicion of pneumonia despite an initially unremarkable chest radiograph and highlights the importance of maintaining a broad differential diagnosis when unexpected respiratory deterioration occurs during treatment.
